# The WHO FCTC’s lessons for addressing the commercial determinants of health

**DOI:** 10.1093/heapro/daab143

**Published:** 2021-12-12

**Authors:** Juliette McHardy

**Affiliations:** O'Neill Institute for National and Global Health Law, 500 First Street NW, Washington DC, 20001, USA

**Keywords:** determinants of health, globalization, governance, tobacco, alcohol

## Abstract

The tobacco, alcohol, beverage, processed food, firearms, gambling, fossil fuel and mining industries, inter alia, are implicated in fostering negative commercial determinants of health. They do this by shaping our environments, tastes, knowledge and politics in favour of the unlimited consumption and unencumbered promotion of their deadly and dangerous products. To shift the determinants of health, emphasis should be put on preventing industry actors whose profit lies in harming health from wielding influence over the institutions and actors of global and national governance. The tobacco control experience and the implementation of the WHO Framework Convention on Tobacco Control (WHO FCTC) provide a unique, comprehensive and fully substantiated guide for how this may be done. Just as the tobacco industry was a pathfinder for other harmful industries in developing tactics for expanding the depth and reach of the market for their deadly products, the WHO FCTC experience is the obvious pathfinder for countering the commercial determinants of health across all sectors and industries. Although they are desirable for countering negative commercial determinants of health, the WHO FCTC’s lesson is not that commercially driven epidemics must be tackled with legally binding treaties. Rather, given the challenges to treaty-making, the key lessons are those that show how it is possible to address the harms of other commodities, even in a treaty’s absence. What is needed is the national implementation of measures providing for intersectoral governance and protection from industry interference which will then assist in unlocking measures for reducing the supply of and demand for unhealthy commodities.

## INTRODUCTION: THE CHALLENGE POSED BY THE COMMERCIAL DETERMINANTS OF HEALTH

### Addressing the commercial determinants of health

The commercial determinants of health (CDoH) are the ‘strategies and approaches used by the private sector to promote products and choices that are detrimental to health’ (Kickbusch *et al.*, 2016). Although some private sector actors may positively influence the determinants of health, this discussion is focused on the lessons from tobacco control and, therefore, only on negative influences. The main non-tobacco industries with actors implicated in fostering negative CDoH include, inter alia, the alcohol, beverage, processed food, firearms, gambling, fossil fuel and mining industries. These industries seek to increase their consumer base and influence our choices in favour of their products by shaping our environments, tastes and knowledge. These choices may directly or indirectly harm our own health and well-being or that of others. Direct harms include diseases and injuries resulting from unhealthy consumption—e.g. respiratory diseases linked to smoking, second-hand smoke, or air pollution or injuries from alcohol-related accidents, suicide and violence (Allen, 2020). Indirect harms include unhealthy labour conditions, toxic or barren environments, intergenerational poverty and climate change (Allen, 2020). In 2019, tobacco, alcohol and sugar-sweetened beverages were alone estimated to have been risk factors for 11.4 million deaths (IHME, 2021).

CDoH harm health to the extent that they are either unprevented or permitted due to a political failure—whether because of incapacity, incomprehension or complicity. CDoH work through influencing, undermining and misdirecting institutions and policymakers who may otherwise confront them. In this way, commercial and political determinants are inextricable. The political determinants of health are constituted by the paradigms, norms and institutions that, in structuring the relationship between government, market and society, define public and private roles in relation to health (Lencucha and Throw, 2019). Private sector influence has conditioned political paradigms, norms and institutions to such an extent that the political and commercial determinants of health are like two sides of one coin.

This commercial power over political determinants accrues through discursive influence that: (i) shifts the blame for negative health effects onto individual choices; (ii) shifts ‘legitimacy’ of a decision from one forum to another as suits industry interests; (iii) exaggerates the economic importance of harmful products; (iv) emphasizes short-term gains over long-term costs; (v) pretends regulation will only work when commercial actors are at the table as ‘part of the solution’ (Fooks *et al.*, 2019; Hessari *et al.*, 2019; Allen, 2020; Lencucha and Throw, 2020). These arguments are often accompanied by industry-funded fake or misleading ‘evidence’ that seeks to minimize health impacts or disguise the relationship with its products (Fooks *et al.*, 2019; Rossow and McCambridge, 2019; Allen, 2020). This discursive influence is underpinned by industry’s financial clout and willingness to use it to co-opt influential persons and organizations as lobbyists and front groups, extract regulatory concessions in return for investment, provide self-interested charity in return for access and, when all else fails, resort to litigation or outright bribery (Hessari *et al.*, 2019; Rossow and McCambridge, 2019; Allen, 2020). Because of this negative dynamic between health’s political and commercial determinants, we increasingly live, work and do politics under conditions in which our health plays, at best, second fiddle to a ‘narrow economic rationality’ (Lencuhca and Throw, 2019).

Fortunately, improving the political determinants of health does not require us to, as some argue, defeat neoliberalism, capitalism or market totalitarianism. During the coronavirus disease 2019 (COVID-19) pandemic, it has become more evident than ever that health and economy are inextricably linked, and that healthy economies are constituted by healthy people (Harper *et al.*, 2021). Actors involved in harmful industries have both minimized their risks to long-term prosperity and exaggerated their indispensability to short-term economic growth (Fooks *et al.*, 2019; Lencucha and Throw, 2019; Allen 2020). It is, accordingly, possible to meet and triumph over health-harming industries on their chosen discursive territory: economic well-being. It is not enough, however, to win a rational argument that industry resources and influence will easily repel.

Accordingly, to shift the political determinants of health, emphasis should be put on preventing industry actors whose profit lies in harming health from wielding influence over the institutions and actors of global and national governance. This is principally done by ensuring, at all levels of government, intersectoral and coordinated governance for health that is bound by specific rules limiting preferences granted to industry actors and requiring all interactions be conducted with absolute transparency (WHO FCTC COP, 2007; WHA, 2014; WHO Shanghai Declaration, 2017; Carriedo *et al.*, 2021). By going on the offensive with supply-side measures, commercial leverage over the political determinants can either be uprooted through measures such as antitrust actions or offset through compensation or alternative support for corporate clients, employees, beneficiaries and dependents. Finally, demand-reduction measures prevent and reverse commercial influence over people, their lived environments and the media they are exposed to.

The tobacco control experience and, in particular, the implementation of the WHO Framework Convention on Tobacco Control (WHO FCTC) provide a comprehensive and fully substantiated guide to countering the CDoH. The needed provisions are set out in this legally binding treaty, in the guidelines for the implementation of some of the articles—adopted with consensus by the Convention’s Conference of Parties (COP)—and in the evidence accumulated over 16 years of regional, national and local implementation. There is not an equivalently comprehensive body of knowledge associated with countering other CDoH. The most developed are those surrounding unhealthy diets and obesogenic environments, but they are generally more descriptive of the industry actors involved and tactics used rather than detailed and prescriptive in relation to the countervailing measures needed (Lacy-Vawdon and Livingstone, 2020). This should not be surprising as tobacco is a longstanding and archetypal harmful commodity, while tobacco industry tactics and the experience in combatting them helped give birth to the CDoH concept (Allen, 2020).

Other industries producing unhealthy commodities, try to distinguish themselves from the tobacco industry and claim that tobacco is uniquely unacceptable; but while other products may not be as deadly as tobacco they are still a significant and comparable source of avoidable morbidity and mortality (Lencucha and Throw, 2020; Lucy-Vawdon and Livingstone, 2020). Similarly, there is strong evidence that tactics used by these other industries, often mirror those of the tobacco industry at both national and global levels (Fooks *et al.*, 2019; Hessari *et al.*, 2019; Rossow and McCambridge, 2019; Allen, 2020; Lacy-Vawdon and Livingstone, 2020; Lencucha and Throw, 2020). Accordingly, just as the tobacco industry was a pathfinder for other harmful industries in developing tactics for expanding the depth and reach of the market for their deadly products, the WHO FCTC experience is the obvious pathfinder for countering the CDoH across all sectors and industries (Allen, 2020; Mialon, 2020).

Although all its provisions need to be drawn upon, this article focuses first, in the upcoming section, on the lessons offered by the Convention’s institutional framework and its provisions on governance. The subsequent section provides guidance on adapting the successes of the WHO FCTC style governance and institutions to the countering of other CDoH before concluding with a discussion of the ambitious urgently needed demand-reduction measures such reform will unlock.

## THE FRAMEWORK FOR ADDRESSING THE CDoH: LEARNING FROM THE WHO FCTC

### The WHO FCTC

**Fig. 1:**
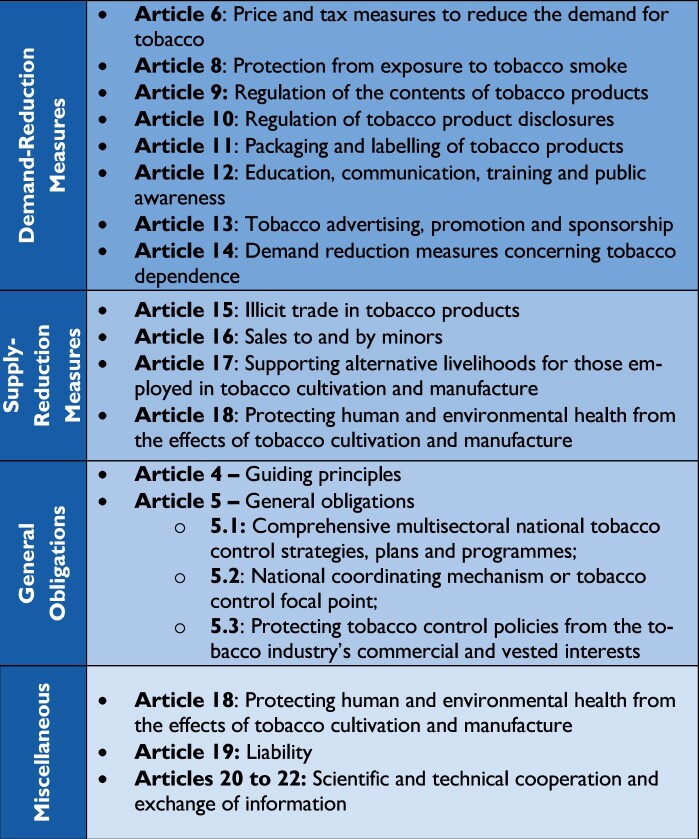
Key provisions of the WHO FCTC.

With 182 Parties, the WHO FCTC’s provisions cover 90% of the global population. Its text (see Figure 1) comprises three broad categories of obligations—governance, supply and demand—as well as other miscellaneous provisions. Sixteen years later, after its 2005 entry into force, the tide is finally turning on the global tobacco epidemic: prevalence has been steadily decreasing and the absolute number of tobacco users, which peaked in 2018, is projected to continue to decline in the years to come (WHO, 2019a). Although demand reduction measures have been its most directly impactful component, the Convention’s successes have been built on the foundation provided by its institutional framework and governance provisions (Zhou and Liberman, 2018).

The WHO FCTC’s governing body is the COP. It comprises all Parties and meets once every 2 years. Its significant normative competencies make the Convention a living instrument able to adapt to changing circumstances and develop in response to ever-evolving tobacco industry tactics. A bedrock for WHO FCTC implementation has been the relationship of mutual cooperation between the WHO FCTC Secretariat (Convention Secretariat) and WHO. The Convention Secretariat, an entity hosted by WHO but established by the Convention, supports the work of the COP and its subsidiary bodies and provides technical assistance to Parties to advance implementation of the WHO FCTC in partnership with WHO and other crucial intergovernmental and non-governmental partners—especially philanthropies, academia and civil society organizations (CSOs).

A key feature of the WHO FCTC is its status as a binding treaty at international law with broad uptake among States. This status fortifies the WHO FCTC’s provisions before legal tribunals and has made the Convention an invaluable instrument in defending domestic and international legal challenges from tobacco industry actors and allies—see Box 1 (Assunta, 2018; WHO FCTC, 2019). It has helped overcome and prevent the deterrent effect that the threat of litigation can pose to policymakers seeking to counter the CDoH (Allen, 2020). It also injects a greater consideration for health into legal institutions and organizations conventionally focused primarily on an economic mandate—such as international economic law’s operational and judiciary bodies (Lenchucha and Throw, 2019). Relatedly, Article 19 provides for the establishment and use of civil and criminal liability measures to hold the tobacco industry accountable for legal violations. Its ground-breaking inclusion in the WHO FCTC has put the otherwise litigious tobacco industry on the defensive in national courts (Box 1).

### Governance

Most tobacco control governance obligations are contained in Article 5 (paragraphs 1, 2 and 3). Paragraphs 1 and 2 are the central provisions of the WHO FCTC’s governance scheme and, arguably, the most innovative aspect of the Convention (Lencucha and Throw, 2020). They require Parties to establish the mandate for national comprehensive multisectoral tobacco control strategies, plans and programmes. They further provide that Parties shall establish or reinforce and finance a national coordination mechanism or a focal point for tobacco control. In this way, the fiction that good governance for tobacco control can be achieved by ministries of health and public health actors alone is denied. This is crucial since many of the most effective interventions, such as taxation, are inherently multisectoral and within the purview of government actors that traditionally regulated tobacco as a source of revenue, employment, foreign exchange, etc. rather than as a threat to health (Fooks *et al.*, 2017; Zhou and Liberman, 2018).

Article 5.3 obliges Parties to protect tobacco control policies from commercial and other vested tobacco industry interests—insulating all policymakers and regulators from tobacco industry influence and making all interactions with the industry transparent. At the Third Session of the COP, Parties adopted the WHO FCTC Guidelines for the implementation of Article 5.3 to ensure its comprehensive, effective implementation by all parts of government interested or involved in tobacco control (WHO FCTC COP, 2007). This intersectoral application across government and throughout its levels not only makes the provision a more effective shield against interference but also has an educational value in ensuring government actors are cognizant of the harms posed by the tobacco industry (Fooks *et al.*, 2017; Lencucha and Throw, 2019). The Guidelines also detail that Article 5.3 is applied not only to the tobacco industry itself, but also to those ‘organizations and individuals that work to further the interests of the tobacco industry’ to ensure against the use of proxies and allies. The Guidelines require transparency which encourages accountability and provides civil society with increased capacity for monitoring tobacco industry attempts to subvert public health goals. The evidence suggests that national initiatives enshrining the independence and transparency of tobacco control policymaking have often preceded and accompanied effective tobacco control—see Box 2 (Assunta, 2018) (Box 2).

The work of two CSOs—the South East Asian Tobacco Control Alliance and the Global Centre for Good Governance in Tobacco Control (GGTC)—on monitoring and tracking tobacco industry interference provides an example of how civil society can help operationalize commitments to counter the CDoH. Their pathfinding tools for monitoring and analysing industry interferences tracks Article 5.3’s national implementation in a standardized format (SEATCA, 2020). The resulting industry interference indexes allow for comparisons of implementation across countries and years to provide snapshots of relative success and track progress over time.

An important aspect of the framework established by the WHO FCTC is its flexibility in the face of ever-evolving tobacco industry tactics. The most prominent example can be seen in the response to novel and emerging tobacco products and nicotine products currently taking root mostly within high-income countries. Although minor as a share of the overall global market, these novel products—heated tobacco products (HTPs) and electronic nicotine and non-nicotine delivery systems (ENDS/ENNDS)—have hijacked discussions on tobacco control policy and created the false impression of fracture within the tobacco control community based on the existence of different approaches towards the regulation of these products.

Meanwhile, the industry has seized the opportunities created by regulatory inertia to circumvent tobacco control regulations and institute its own version of ‘harm reduction’. While harm reduction is a well-known strategy used to decrease the individual and public health burden of, for example, use of illicit drugs, the tobacco industry only uses the concept to expand the nicotine market while rehabilitating its public image (see Box 3). This is evident in the targeted marketing of ENDS and HTPs to youth, instead of the industry’s purported target market of medium-aged smokers who cannot quit using established and evidence-based cessations techniques (Box 3).

Unfortunately, the value of any ‘innovation’ coming from the tobacco industry remains to be scientifically proven and cannot be presumed given the deceptions of the 1960s through to 1990s with many iterations of ‘safer products’—most prominently with the ‘light’ ‘low-tar’ cigarette lie (Minhas and Bettcher, 2010; NCI and WHO, 2016; Bialous and Glantz, 2018; Bialous, 2019; Evan-Reese *et al.*, 2020). As occurred with ‘light’ cigarettes, in the marketing material and public pronouncements of all leading industry actors you can see aspirational pledges to a ‘smoke-free future’, for ‘a better tomorrow’, etc. In reality, the industry is aiming first and foremost to undermine tobacco control and put a gloss on the ongoing and undiminished sale of their regular, deadly tobacco products across the world (Bialous and Glantz, 2018; Evan-Reese *et al.*, 2020; National Academies of Science, Engineering and Medicine, 2018).

Because of its institutional capacity, the WHO FCTC has been able to meet these emerging challenges. The Seventh Session of the COP adopted a decision on ENDS and ENNDS inviting Parties to regulate these devices by prohibiting or restricting their manufacture, importation, distribution, presentation, sale and use (WHO FCTC COP, 2016). The Eight Session of the COP adopted, in turn, a decision on novel and emerging tobacco products, recognizing that HTPs ‘are tobacco products and are therefore subject to the provisions of the WHO FCTC’ (WHO FCTC COP, 2018).

### Indicators, surveillance and data for driving implementation

The tobacco industry has the resources to sponsor and fabricate the evidence it needs to obstruct regulation by minimizing health impacts, creating a fear of unintended effects and, most significantly, reframing tobacco control as a threat to economic well-being (Bialous, 2019). This tactic is designed to win over finance, labour and agriculture ministries already sceptical of arguments on health due to an institutional tradition of construing tobacco only as an issue of revenue, employment or exports. Lacking the wherewithal or prioritization needed to counter this ‘evidence’, health ministry policymakers often have no choice but to accept tobacco industry assertions and substantiation of an economic effect at face value and instead argue only on the grounds of impact to health—a weak ground to other government sectors. Public health actors—including the Convention Secretariat, WHO, the World Bank and CSOs—have acted to counter this tobacco industry machination by using surveillance and collating this data into generalized and country-specific arguments for tobacco control.

The most prominent example of this is the MPOWER technical package. Introduced in 2008 by WHO in collaboration with key partners in the Bloomberg Initiative to Reduce Tobacco Use, it both supports and tracks country-level implementation of the WHO FCTC’s demand reduction measures (see Figure 1, above). Country progress on the MPOWER measures is collated and published every 2 years in the biennial WHO Global Report on the Tobacco Epidemic (known as GTCR) alongside comprehensive statistics on manifold other aspects of tobacco control. The scope and depth of the GTCR’s data have liberated policymakers and the tobacco control community from their former reliance on tobacco industry produced statistics (for an example, see Box 4). WHO has also measured and quantified the cost-effectiveness of the WHO FCTC’s demand reduction measures as ‘Best Buys’ that provide a high return on investment—contained in the NCD Global Action Plan (NCD GAP). Together they provide evidence with which policymakers can counter industry claims that tobacco control will harm the economy (Box 4).

## THE WHO FCTC’S LESSONS FOR OTHER CDoHS

The WHO FCTC’s success has inspired calls for treaties to address other NCD risk factors as well as other CDoH (Burci, 2018). This makes sense as treaties have the advantage of establishing obligations binding at international law which can be overseen by a governing body and a permanent secretariat. Beyond their legally binding nature, processes of negotiation and ratification needed for treaty-making confer credibility and legitimacy on the resultant norms that impel and facilitate implementation (Burci, 2018). Further, when a framework approach is used, the principal treaty obligations can be added to subsequently with subsidiary protocols on specific topics—as occurred with the Protocol to Eliminate Illicit Trade in Tobacco Products adopted by the COP in 2012, now in force since 2018 (Zhou and Liberman, 2018). Citation of a treaty obligation can also be highly persuasive in legal tribunals when defending a measure to counter the CDoH—as seen with Uruguay (Box 2) in which the WHO FCTC proved of paramount importance in defending such a measure (Burci, 2018; Zhou and Liberman, 2018; Nikogosian and Kickbusch, 2018).

At the same time treaties have two main disadvantages. The first is the risk that negotiations can fail or result in only an unworkable or ineffective treaty (Hoffman *et al.*, 2015). The second is cost: negotiations come with high-transaction costs and any benefits will only be realized relatively far in the future (Hoffman *et al.*, 2015). Even for the WHO FCTC—which was negotiated, ratified and entered into force in record time—over a decade elapsed from 1993, when the idea was first proposed by two academics, Ruth Roemer and Allyn Taylor, to its actual entry into force in 2005 (Roemer *et al.*, 2005). The WHO FCTC’s success has also likely increased the challenges of negotiating a treaty to tackle commercially driven epidemics as it may have stiffened the resolve of other industries to resist international regulation and informed their strategies of interference (Burci, 2018).

Many of the benefits that international obligations can bring are also accessible without a treaty because, at the end of the day, a treaty is effective only if its mandates are incorporated in national legislation and it is possible to legislate for most such mandates in the absence of any treaty (Hoffman *et al.*, 2015; Burci, 2018). For example, the system for intersectoral governance and countering industry interference established by Uganda to implement the WHO FCTC (see Box 2) could be established by any country in relation to any other CDoH without the existence of a related treaty. Similarly, health protective legal norms can be internationalized without a standalone treaty through their insertion into other international legal regimes, for example, in negotiations on free trade or international investment agreements (Burci, 2018; Zhou and Liberman, 2018). For example, there would have been no investment treaty claim against Uruguay (see Box 1) if the relevant treaty had accorded special status to health protective measures such as tobacco control or, as should be the model, health promoting measures in relation to any and all CDoH. It is also possible for States to create and operationalize systems for accountability and transparency that track and guide implementation without a treaty by assigning these functions to an existing institution such as WHO—as occurs with the NCD GAP, discussed below (Hoffman *et al.*, 2015).

Although treaties would be ideal if there were no concerns for timeliness, cost and capacity, there is empirical evidence to suggest that alternative soft-law norms for supporting national action and international cooperation can be as effective and well complied with as those of hard-law treaties given the correct circumstances (Burci, 2018). A key question is how credible and legitimate the process for a soft-law instruments adoption is perceived to have been (Burci, 2018). While it is unlikely that any could rival the WHO FCTC in credibility and legitimacy—partly because the Convention is an exceptionally widely adopted and well-implemented treaty that cannot be considered typical—there are existing precedents for the adoption of soft-law instruments with processes that are comparably credible and legitimate. For example, targets, standards and plans of action for addressing a real issue in global public health with solutions based on evidence and extensive consultation adopted by resolutions of the World Health Assembly (WHA) or the United Nations General Assembly. The resultant soft-law instruments are perceived as credible and legitimate because of the evidence, process and actors involved.

This can be seen in existing models for addressing aspects of the CDoH with alternative soft-law instruments that are effective at promoting national implementation of key demand-reduction measures for other harmful commodities despite not being contained in a legally binding treaty (Burci, 2018; Garde, 2018). For example, the NCD-GAP’s Best Buys and Good Buys have soft-law authority stemming from their adoption by the WHA. This authority is reinforced by their status as a precise, evidence-based and authoritative statement on the measures needed for prevention and control of NCDs equivalent to MPOWER (Garde, 2018). An important additional feature is that WHO reports back to the WHA on progress towards the non-binding goals set out in the NCD-GAP providing a monitoring structure that proved useful for motivating and assessing progress in the WHO FCTC’s implementation. Another example of a soft alternative to treaty-making is the report of the WHO’s Commission on Ending Childhood Obesity (ECHO report). It comprises approaches and interventions that the evidence suggests will be effective in tackling childhood and adolescent obesity in different national contexts (Garde, 2018).

### Essential aspects: governance structures

Article 5 of the WHO FCTC is of paramount importance to understanding the Convention’s success (Garde, 2018; Zhou and Liberman, 2018; Lenchucha and Throw, 2020). Paragraph 3 of Article 5 is the most strikingly distinct aspect of the tobacco control experience compared with prevailing approaches to countering other CDoH. Its rigorous protection of public policies for tobacco control has not been replicated in approaches to the regulation of other industries. It is, however, as has been noted, possible for the model to be adapted and implemented nationally for other CDoH without there being equivalent binding treaties. While the quasi-absolute restriction on interactions with industry urged by Article 5.3’s guidelines may not, in all cases, be necessary for other risk factors, the fundamental principles of transparency and public policy protection are universally applicable (Fooks *et al.*, 2017; Garde, 2018).

Commercial actors responsible for producing, promoting and selling alcohol, processed foods and sugary beverages are commonly able to access governments with as much ease as other economic actors and often with special preferences (Allen, 2020; Carriedo *et al.*, 2021). Although there are success stories, such engagement comes with the risk of regulatory capture, subversion of public health goals and substitution of weak or voluntary measures for the strong mandatory regulation actually needed (Garde, 2018; Brown, 2019; Allen, 2020; Mialon, 2020; Mialon *et al.*, 2020a, 2020b; Carriedo *et al.*, 2021). According to a survey by a leading CSO, the NCD Alliance, of its national and regional affiliates, only 7% of respondents believed their government(s) had sufficient mechanisms for preventing even basic conflict of interest issues with the food, beverage and alcohol industries (Renshaw *et al.*, 2020).

One answer to these defects is to require that public health purposes be served—rather than determined—by interaction with commercial interests. Another is to require that even an ostensibly beneficial engagement be transparent, fully disclosed and, where possible, held publicly while also ensuring private industry engagement is paired with civil society engagement. All regulatory bodies and public officials should also be protected from gratuitous interactions with industry actors by clear, codified and enforced conflict of interest rules—to ensure otherwise useful engagement does not become a lobbying opportunity.

As occurred with the WHO FCTC’s implementation, reforms enshrining intersectoral coordination and the protection of health policymaking can be implemented at the national level through either administrative directives or legislation (Fooks *et al.*, 2017; Assunta, 2018). Notwithstanding the lack of a global treaty on alcohol, sugary beverages or unhealthy foods, there is no reason why such reforms will not be as successful as those for implementing Article 5 (Fooks *et al.*, 2017). Excepting actual violations of national law, including bribery and illicit trade, by multinational corporations, there are not any significantly transboundary or cross border aspects to preventing industry interference for these CDoH that would make having a treaty indispensable (Hoffman *et al.*, 2015; Fooks *et al.*, 2017; Burci, 2018). But the continued under-implementation of the WHO FCTC’s Article 5 provisions also shows that these reforms are difficult to realize and sustain over time due to both inertia and political entropy in the face of industry resistance and shifting governmental priorities. Because of this, where treaty-making is not practicable, there is a strong case for establishing soft-law instruments in support of intersectoral coordination and the prevention of industry interference as well as systems of international support for their implementation.

Relatedly, another key lesson of the WHO FCTC’s implementation is the need to support measures for countering the CDoH at the country-level with both generally applicable and specific arguments based on scientific evidence—for example, data on consumption patterns and disease epidemiology or modelling on the likely effects of a measure (Fooks *et al.*, 2019; Allen, 2020). These resources are essential for overcoming information and resource disparities between governments and well-funded multinationals (Fooks *et al.*, 2019; Rossow and McCambridge, 2019; Allen, 2020). For example, just as Phillip Morris International funded FSFW (see Box 3) to promote ‘harm reduction’, Coca-Cola surreptitiously funded the ‘Global Energy Balance Network’ to argue that insufficient exercise is the more important risk factor for obesity (Hessari *et al.*, 2019; Allen, 2020). In the absence of the resources described above, governments will find it more difficult to hold companies to account and oppose the self-interested claims that industry actors make in their lobbying.

An interesting example of support that serves this purpose for other CDoH is the relatively novel Corporate Permeation Index (Lima and Galea, 2019). It goes beyond tobacco industry interference while remaining more specific than a mere measurement of perceived public sector corruption by examining the extent to which corporations can influence society and government through both legitimate and illegitimate means (Lima and Galea, 2019). The authors specifically expect it to be of use in identifying the relative presence and effect of commercial determinants of alcohol consumption, obesogenic diets and other risk factors both between countries and within single countries over time (Lima and Galea, 2019). Another example is noted below, at the end of this section, in relation to health taxes.

### Essential aspects: demand reduction measures

Protecting public health policymaking from industry interference is usually necessary but by itself insufficient for countering the CDoH. It is, rather, a preliminary step that makes other essential and necessary action possible. When it comes to the alcohol, food and beverage industries, in particular, it is also crucial for measures to be implemented that counteract their direct influence over people with demand-reduction measures (Garde, 2018). As with the WHO FCTCs implementation, it is important that policymakers consider and implement demand-reduction measures for other harmful commodities as a system of complementary interventions that are more effective when coordinated with one another (Garde, 2018; Zhou and Liberman, 2018).

The marketing—including advertising, promotion and sponsorship—of alcoholic beverages, unhealthy foods and sugary beverages should be regulated to limit the exposure of people, and in particular vulnerable groups, such as children, to industry influence (Garde, 2018). Applicable regulation can and often should amount to complete marketing bans—as is required for tobacco under analogous provisions of the WHO FCTC, Articles 11 and 13. For alcohol, in particular, marketing restrictions are highly cost-effective ‘Best Buys’ for preventing harm to human health in both high-income and low-income settings (WHO, 2013; Chrisholm *et al.*, 2018). For unhealthy foods and beverages, marketing restrictions are substantiated as particularly effective in protecting children from obesity (Allen, 2020; Ryan *et al.*, 2020). Such protection requires a comprehensive set of measures that can target the various media through which commercial industry influence over children is channelled. For example, in Chile, the marketing of various harmful products is entirely banned from certain media children may be exposed to and the use child-friendly presentation, such as cartoon characters, is restricted (Ryan *et al.*, 2020). There is also a need to adapt to the digitization of the CDoH. A pathfinder for a cutting-edge response is WHO’s use of artificial intelligence to track and respond to COVID misinformation. The same technology should be used to directly counter the CDoH by repurposing the online channels used by industry to distribute evidence-based information tailored to respond to the industry’s marketing messages.

Corporate social responsibility (CSR), charitable gifts to selected causes, from harmful industries should be limited or banned entirely to prevent its use as a combined public relations and marketing campaign (Allen, 2020). For example, food and beverage industry actors use purportedly charitable expenditure to fund sporting events and infrastructure for community exercise to distract for the harm of their products, emphasize the importance of physical inactivity, and associate their products with aspirational active people and events (Hessari *et al.*, 2019; Allen, 2020). Similarly, alcohol industry actors or associations fund campaigns to encourage ‘responsible’ drinking that place the onus on individual choice and distract from their role as the sellers and marketers of this harmful and addictive product (Yoon and Lam, 2013). Importantly, by banning CSR, governments will be removing a mechanism by which industry actors seek to divert small tax-deductible portions of their revenue into substantial influence on key political constituencies and policymakers as well as indirect marketing to the general public (Yoon and Lam, 2013; Allen, 2020).

Nutrition and warning labels should be included on alcohol products as well as unhealthy foods and beverages to ensure people are informed of the risks posed—offsetting industry marketing tactics that minimize or divert attention away from risks. Substantial evidence now confirms that, warning labels are effective for reducing demand for unhealthy foods and beverages and there is similar evidence on the using of warnings for alcoholic beverages (Ryan *et al.*, 2020). As seen in the WHO FCTC’s implementation (see Box 1), to be more effective and to reach people without strong health literacy, such as the young, these warnings need to be made visible and impactful (Garde, 2018). For example, in 2016, Chile required front of pack warning labels—standardized sizable warnings against visible black backgrounds—for products high in sodium, sugar, calories, saturated fat and trans fat (Ryan *et al.*, 2020). Substantial reductions, 14% and 25%, respectively, were reported in the purchases of sugary breakfast cereals and sugary beverages (Ryan *et al.*, 2020). The Chilean model has seen widespread adoption and deserves consideration as a best practice for countering the CDoH of obesity. There is no reason why similar highly visible warnings should not be required for alcohol products but at the very least packaging should feature comprehensive warnings of alcohol’s health risks (Kaczmarek, 2017). Despite this, mandatory warnings on alcohol products are reasonably rare and even when required frequently provide incomplete information in low-visibility and text-only warnings (Kaczmarek, 2017; May *et al.*, 2021).

As with marketing, availability restrictions—that limit the physical accessibility of harmful products—are highly cost-effective and should be required for alcohol, unhealthy foods and sugary beverages. Industry actors seek to make their products as accessible as possible and the harm from this CDoH can only effectively be reduced with regulation. In terms of alcohol, restrictions on the availability of alcohol via reduced hours of sale is a highly cost-effective ‘Best Buy’ but can and should be paired with other recommended restrictions on availability such as those that limit the density of outlets selling alcoholic products and provide for a minimum age of purchase (WHO, 2013; Chrisholm *et al.*, 2018; Rossow and McCambridge, 2019; Rehm *et al.*, 2020). For dietary risk factors, restrictions on availability are generally more targeted with most measures focused on protecting children and making school environments more health promoting. Brazil is a trailblazer with requirements that 30% of foods offered in school come from local farmers and evidence that the requirement has prompted many schools to exceed the requirement by as much as two-fold (Ryan *et al.*, 2020). Other countries have restricted or banned the sale of sugary beverages on the premises of schools (Bergallo *et al.*, 2018).

A key lesson of the tobacco control experience is that using health promoting taxation (‘health taxes’) to reduce the affordability of unhealthy products is a highly effective and cost-effective way of reducing demand and harm (Garde, 2018). Alcohol and sugary beverage taxes are, respectively, a ‘Best Buy’ and a ‘Good Buy’ for NCD prevention (WHO, 2013). Taxation measures to discourage consumption of unhealthy foods are also recommended in the WHO ECHO Report (WHO, 2018; PAHO, 2020). Despite the rationales and evidence in their favour, health taxes on commodities other than tobacco are relatively underutilized.

This is in part due to a comparative lack of support that addresses the political economy of health taxation. To address this, WHO has already leveraged its existing expertise on tobacco taxes to create a *fiscal policies for health unit* capable of supporting governments in taxing alcohol and sugary beverages by facilitating the production of technical guidance, political economy analyses and reliable data. Similarly, the non-governmental Taskforce on Fiscal Policy For Health co-chaired by Mike Bloomberg and Larry Summers, as well as supported by Bloomberg Philanthropies, have contributed to this support by developing a global investment case—in terms of lives saved, revenue raised and the distributional benefits for lower-socioeconomic groups—for the taxation of sugary beverages, alcohol and tobacco (Task Force on Fiscal Policy for Health, 2019). These efforts and those like them help counteract the CDoH by revealing the falsehoods, simplifications and exaggerations that underpin industry arguments against health promoting measures such as health taxes (Fooks *et al.*, 2019; Carriedo *et al.*, 2021).

To tackle the CDoH for alcohol and unhealthy diets, countries need to act on the evidence, draw on this growing support and be bold in introducing effective health taxes. Particularly instructive is the last decade of experimentation with the taxation of sugary beverages and the rapidly accumulating evidence of success. In 2014, Mexico introduced a sugary beverage tax that within a year resulted in a 7.6% reduction in purchases (Ryan *et al.*, 2020). Later studies have shown the reductions in purchases of sugary beverages to have been sustained 2 years following the tax’s introduction (PAHO, 2020) The UK also introduced a sugary beverage tax but, unlike Mexico’s, its measure targeted beverages with higher sugar contents with much higher tax burdens. Within 2 years, there was an 11% reduction in the sugar content of taxed sugary beverages due to reformulation by manufactures and the volume of high-sugar beverages sold fell 40% due to the combined effect of reformulation and demand-reduction caused by higher prices (PAHO, 2020).

## CONCLUSION

Although legally binding treaties addressing other CDoH—or even an overarching treaty on countering the CDoH—may be desirable and should be pursued when it is likely that their benefits will outweigh their costs, the essential lesson of the WHO FCTC is that, even in a treaty’s absence, certain national measures providing for intersectoral governance and protecting public policy from industry interference will still assist in unlocking needed measures for reducing the supply of and demand for unhealthy commodities. There is an urgent need for this lesson to be taken up and applied for addressing the harms of alcohol, sugar beverages and unhealthy foods. It is, however, also directly applicable to other demand driven threats to user health, such as gambling, mediated by the CDoH while being adaptable to countering the CDoH of other risk factors. While this approach does not exclude all opportunities for collaboration with actors from non-tobacco industries, it does support evaluating and regulating engagement with actors from industries with proven records of contributing to the negative CDoH. This perspective is supported by a recently published Lancet Paper, which shows that NCD prevention policies are less well implemented in countries that have had less success in improving the CDoH (Allen *et al.*, 2021).

**Box 1: Case study 1: Uruguay**

Uruguay has successfully relied on the legal support of the WHO FCTC to defend its leadership in the implementation of demand reduction measures and, in particular, its mandating of effective warnings on the harms of tobacco (Zhou and Liberman, 2018). This began in 2009 with efforts to implement Articles 11 and 13 of the WHO FCTC through increases in the size of graphic health warnings to cover 80% of cigarette packages and a single presentation per family brand requirement. In 2010, Philip Morris responded with a legal challenge under a bilateral investment treaty between Switzerland and Uruguay. In July 2016, all of the challenges were dismissed (Phillip Morris v Uruguay, 2016).

In finding that there had been no breach, the arbitral tribunal relied heavily on submissions—by the Pan-American Heath Organization, WHO, and WHO FCTC Secretariat—which provided evidence on the tobacco industry’s long history of interference and substantiated Uruguay’s argument that the measures were reasonable, evidence-based, effective and implemented as part of a duty to protect public health (Phillip Morris v Uruguay, 2016). In coming to this conclusion, the WHO FCTC and the COP guidelines to its provisions were relied on as both authorities and authoritative encapsulations of the evidence base (Phillip Morris v Uruguay, 2016).

**Box 2: Case study 2: Uganda**

In 2015, Uganda sought to insulate itself from industry interference by mandating intersectoral action on tobacco control and transposing Article 5.3 and its Guidelines directly into national legislation—the Tobacco Control Act (2015) (Assunta, 2018). The locus of the Act is its oversight by a high level Tobacco Control Committee comprising Ministry of Health officials, CSO representatives and Office of the Prime Minister officials (Assunta, 2018). The Committee advances an intersectoral approach to tobacco control policy and the prevention of commercial interference via consultation across government (Assunta, 2018). This implements the WHO FCTC guidance to Article 5.3 by limiting interactions with tobacco industry and requiring their transparency while prohibiting the receipt of contributions from or granting of incentives to the tobacco industry (Assunta, 2018). All these prohibitions extend beyond the government and its employees to other people and actors that may be involved with tobacco control policies—see the Tobacco Control Act (2015) sections 20–23, 25. The extent to which the Act refers to and mirrors Article 5 and the guidance to Article 5.3 demonstrates how the credibility and legitimacy of the WHO FCTC has impelled and structured progress on tobacco control.

While further study is needed, the impact of Uganda’s intersectoral approach to counteracting commercial influence is demonstrated in its ranking as the third least interfered with country in the GGTC’s Global Index for 2020 (GGTC, 2020). This relative success has come despite the tobacco industry’s use of economic and legal coercion in an attempt to prevent and then void the 2015 Act. Prior to the Act’s passage, British American Tobacco (BAT) claimed that they would have to cease operations in Uganda because of the effect of the Act on demand, despite the fact that most of the country’s crop was exported (Gilmore *et al.*, 2015). After the Act was passed, BAT launched legal challenges alleging that the rules to prevent industry interference were discriminatory (BAT Uganda v. Attorney General, 2019). This challenge was rejected at all levels of the court system before being definitively dismissed at the Constitutional Court in 2019 as baseless and diversionary interference with tobacco control (BAT Uganda v. Attorney General, 2019).

**Box 3: Case study 3: The foundation for a smoke free world**

A component of the ‘harm reduction’ strategy is the Foundation for a Smoke Free World (FSFW) entirely funded by Phillip Morris International. This is an example of the industry’s self-interested ‘innovation’ being laundered into ‘science’. Provided an annual budget of US$80 million, it purports to espouse a a goal of advancing tobacco control and cessation through ‘harm reduction’ and ‘industry transformation’ when it, in fact, amounts to little more than a legitimising veil for disbursing industry money to favoured sympathetic researchers and for industry public relations messaging. This blatant attempt to evade and subvert article 5.3 of the WHO FCTC is emblematic of the broader strategy behind the narrative of ‘harm reduction’ contrived by the tobacco industry to spearhead their novel and emerging tobacco and nicotine products. The resilience and efficacy of the intersectoral approach and clear red lines established by the Convention have been demonstrated by this episode with nearly all intergovernmental, governmental and credible non-governmental actors refusing any association with FSFW. This rejection is entirely the result of its funding structure and clear position as a proxy for the tobacco industry rather than a judgement on its specific advocacy points.

**Box 4: Case study 4: Tax data, modelling and implementation**

MPOWER introduced two metrics for measuring taxation implementation: (i) the proportion tax represents the average retail price of a package of 20 cigarettes of the most sold brand; and (ii) affordability in terms of GDP per capita required to purchase 2000 cigarettes of the year’s most sold brand (WHO, 2019b). Together, these indicators represent credible targets for best-practice tobacco taxation that adapt to circumstances and motivate the WHO FCTC’s implementation.

Equally important is that, because of the GTCR, WHO’s tax and price data—which include other indicators such as tax structure, tax administration and earmarking—are unrivalled in their breadth, depth and quality with no comparable effort yet existent for other taxed unhealthy commodities, such as alcohol and sugary beverages. Among other things, such data increase the accuracy with which the price, demand-reduction and revenue effects of tax increases can be modelled. It is crucial to emphasize that this contribution is valuable because good governance is built on access to reliable data. When available, it provides governments the ability to choose more cost-effective options for creating healthier environments and provides evidence-based arguments for countering the pressure of commercial interests.
